# *In vitro* antimicrobial activity and resistance mechanisms of the new generation tetracycline agents, eravacycline, omadacycline, and tigecycline against clinical *Staphylococcus aureus* isolates

**DOI:** 10.3389/fmicb.2022.1043736

**Published:** 2022-11-22

**Authors:** Weiliang Zeng, Xiaotuan Zhang, Yan Liu, Yi Zhang, Mengxin Xu, Sipei Wang, Yao Sun, Tieli Zhou, Lijiang Chen

**Affiliations:** ^1^Key Laboratory of Clinical Laboratory Diagnosis and Translational Research of Zhejiang Province, The First Affiliated Hospital of Wenzhou Medical University, Wenzhou, China; ^2^School of Laboratory Medicine and Life Science, Wenzhou Medical University, Wenzhou, China

**Keywords:** eravacycline, omadacycline, tigecycline, *Staphylococcus aureus*, antimicrobial activity, resistance mechanisms

## Abstract

In this study, we investigated the *in vitro* activity and resistance mechanisms of the new generation tetracycline agents, namely eravacycline, omadacycline, and tigecycline, against *Staphylococcus aureus* isolates. A total of 1,017 non-duplicate *S. aureus* isolates were collected and subjected to susceptibility testing against eravacycline, omadacycline, and tigecycline using the broth microdilution method. Tetracyclines-resistant (eravacycline/omadacycline/tigecycline-resistant) isolates were selected to elucidate the resistance mechanisms using polymerase chain reaction (PCR), cloning experiment, efflux pump inhibition, and quantitative real-time PCR. The results of the antibacterial susceptibility testing showed that compared with omadacycline, eravacycline and tigecycline had superior antibacterial activity against *S. aureus* isolates. Among 1,017 *S. aureus*, 41 tetracyclines-resistant isolates were identified. These resistant isolates possessed at least one tetracycline resistance gene and genetic mutation in the MepRAB efflux pump and 30S ribosome units. A frameshift mutation in *mepB* was detected in most tetracyclines-resistant strains (except for JP3349) compared with tetracyclines-susceptible (eravacycline/omadacycline/tigecycline-susceptible) strains. This was first shown to decrease susceptibility to omadacycline, but not to eravacycline and tigecycline. After treatment with eravacycline, omadacycline or tigecycline, overexpression of *mepA*, *tet38*, *tet*(K) and *tet*(L) was detected. Moreover, multi-locus sequence typing showed a major clonal dissemination type, ST5, and its variant ST764 were seen in most tetracyclines-resistant strains. To conclude, eravacycline and tigecycline exhibited better activity against *S. aureus* including tetracycline-resistant isolates than omadacycline. The resistance to these new generation tetracyclines due to an accumulation of many resistance mechanisms.

## Introduction

*Staphylococcus aureus* is an important nosocomial pathogen that is associated with various infections, such as skin and skin structure infections, endocarditis, and bloodstream infections (Pérez-Montarelo et al., 2018). *S. aureus* is one of the major and most fatal causes of bacteremia, and has a mortality rate of almost 20%. Almost half of the patients with *S. aureus* bacteremia will develop complicated bacteremia (Guimaraes et al., 2019). Recently, the emergence of multidrug-resistant (MDR) superbugs, such as methicillin-resistant and vancomycin-resistant *S. aureus*, has become a significant threat to public health (Gasch et al., 2014; McGuinness et al., 2017). The methicillin-resistant *S. aureus* (MRSA) is considered a prioritized nosocomial pathogen by the World Health Organization (WHO; Serral et al., 2021). Overall, *S. aureus* infections are considered a significant clinical challenge.

The tetracycline class of antimicrobial agents has been clinically used for more than 60 years (Roberts, 2003). And they are continued to treat various serious infections caused by Gram-positive and-negative pathogens, including MRSA (Grossman, 2016). With their extensive use in clinical settings, resistance to old tetracyclines, especially doxycycline and tetracycline, is increasing worldwide (Roberts, 2003, 2005; Grossman, 2016). New generation tetracycline agents, such as eravacycline, omadacycline, and tigecycline, can be used as a treatment option for bacterial infections owing to their broad-spectrum antimicrobial activity.

Tigecycline is a semisynthetic glycylcycline with broad-spectrum antimicrobial agents (Doan et al., 2006). In 2005, it was approved by the US Food and Drug Administration (FDA) approval for the treatment of various serious infections, such as adults with complicated intra-abdominal infections and community-acquired bacterial pneumonia (Noskin, 2005). Eravacycline and omadacycline with modifications at the C-9 position are newer generation tetracycline agents similar to tigecycline. They were approved by the FDA in 2018 for the treatment of polymicrobial MDR infections (Solomkin et al., 2019; Morrissey et al., 2020; Zhanel et al., 2020). Similar to other tetracyclines, these new generation tetracycline agents inhibit bacterial protein synthesis by binding to the 30S ribosomal subunit. Eravacycline, omadacycline and tigecycline have broad-spectrum antibacterial activity against Gram-positive and Gram-negative microorganisms. However, the antimicrobial activity of these tetracyclines against *S. aureus* has not been comprehensively studied.

Many mechanisms of underlying tetracyclines resistance have been proposed. The resistance is mostly due to the acquisition of tetracycline resistance genes and mutation of ribosomal protection proteins (Nguyen et al., 2014; Beabout et al., 2015; Grossman, 2016; Linkevicius et al., 2016). Overexpression of efflux pumps has been reported both in Gram-positive and Gram-negative pathogens (Truong-Bolduc et al., 2015; Fiedler et al., 2016; Zhanel et al., 2016). Mutations or overexpression of the MepRAB efflux pump contributes to the decreased susceptibility to tigecycline (McAleese et al., 2005; Fang et al., 2020). Recent studies have revealed that a branched-chain amino acid transport system II carrier protein affects eravacycline and omadacycline susceptibility in *S. aureus* (Bai et al., 2019; Wang et al., 2020). Moreover, a recent study revealed a novel *tet*(L) efflux pump that confers resistance to eravacycline and tigecycline resistance in *Staphylococcus* (Wang et al., 2021). However, the potential contribution of these resistance factors of tetracyclines to the development of resistance to eravacycline and omadacycline in *S. aureus* is not completely known. In addition, multi-locus sequence typing (MLST), which was first established by Maiden et al. (1998), is now the most frequently used method to monitor epidemiology and investigate evolution of pathogens due to its high discriminatory power and comparability (Li T. et al., 2022). The distribution of sequence types (STs) profiles was unclear in tetracyclines-resistant (eravacycline/omadacycline/tigecycline-resistant) *S. aureus* Taken together, the results of this study will give insight into the prevalence and molecular epidemiology characteristics of tetracyclines-resistant *S. aureus* using MLST method.

In this study, we investigated and compared the *in vitro* antimicrobial efficacy of eravacycline, omadacycline, and tigecycline against 1,017 clinical *S. aureus* isolates. Resistance determinants and STs profiles of the tetracyclines-resistant isolates were further investigated using polymerase chain reaction (PCR). Furthermore, genetic mutations in the MepRAB efflux pump and 30S ribosome units and expression of *mepA*, *tet38*, *tet*(K) and *tet*(L) were also determined by sequencing, cloning experiment, efflux pump inhibition and quantitative real-time PCR (RT-qPCR).

## Materials and methods

### Bacterial isolates and plasmids

This study used strains obtained from the First Affiliated Hospital of Wenzhou Medical University. The First Affiliated Hospital of Wenzhou Medical University of institutional ethics committee did not require the study to be reviewed or approved by an ethics committee as this study, which was of observational nature, mainly focused on bacteria and did not involve any interventions to the patients.

From January 2018 to December 2020, a total of 1,017 non-duplicate clinical *S. aureus* isolates, including 577 tetracycline-resistant and 440 tetracycline-susceptible strains, were obtained from the First Affiliated Hospital of Wenzhou Medical University, Zhejiang, China. These bacteria were identified by matrix-assisted laser desorption/ionization time of flight mass spectrometry. *S. aureus* ATCC 29213 and *Escherichia coli* ATCC 25922 were used as quality control strains in antimicrobial susceptibility testing experiments. *E. coli* strain DH5α and pUCP24 plasmid were used as a recipient and vector in cloning experiments (Cheng et al., 2020), respectively.

### Antimicrobial susceptibility testing

The minimum inhibitory concentration (MIC) of eravacycline, omadacycline, and tigecycline was determined by the broth microdilution method. The results were interpreted in accordance with a published research (Zhao et al., 2019), and the European Committee on Antimicrobial Susceptibility Testing, and FDA (FDA, 2020). The tetracyclines-resistant isolates were selected to evaluate their resistance mechanisms. Moreover, the resistance spectrum was examined using the agar dilution method among the resistant strains and ten tetracyclines-susceptible (eravacycline/omadacycline/tigecycline-susceptible) strains randomly selected, including ciprofloxacin (CIP), gentamicin (GEN), levofloxacin (LVX), erythromycin (ERY), linezolid (LNZ), oxacillin (OXA), rifampicin (RIF), and vancomycin (VAN). The interpretation criteria of MIC following the CLSI guidelines (CLSI, 2022). Antimicrobial susceptibility testing was repeated in triplicate. Eravacycline and omadacycline were obtained from the MCE company (Med Chem Express, Monmouth Junction, NJ, United States), and other antibiotics were purchased from Kangtai Biotechnology company (Wenzhou, China).

### PCR detection of tetracycline resistance genes, MepRAB efflux pump-encoding genes and 30S ribosome subunits mutations

Genomic DNA of tetracyclines-resistant strains, ten tetracyclines-susceptible strains randomly selected, and *S. aureus* ATCC 29213 were extracted using the Biospin Bacterial Genomic DNA Extraction Kit (Bioflux, Tokyo, Japan). The *tet*(K), *tet*(L), *tet*(M), *tet*(O), and *tet*(S) genes were screened by PCR amplification with specific primers (Supplementary Table S1). Mutations in the MepRAB efflux pump-encoding genes (*mepR*, *mepA*, and *mepB*) and 30S ribosome protein (*S3* and *S10*) were detected by PCR, and the positive PCR products were sequenced by the Shanghai Genomics Institute Technology Co. Ltd. Then, genetic mutations were analyzed by comparison with the genome of *S. aureus* ATCC 29213.

### Cloning experiments of *mepB*

Based on the PCR data, JP3936 (omadacycline-resistant and eravacycline/tigecycline-susceptible, with a frameshift mutation in *mepB*), JP4612 (tetracyclines-resistant, with a frameshift mutation in *mepB*), and JP4200 (tetracyclines-susceptible, without a frameshift mutation in *mepB* gene) were selected in the experimental and control groups of cloning experiments, respectively. As described in a past study (Cheng et al., 2020), for cloning, *EcoR*I (Takara) and *Xba*I (Takara) restriction endonuclease sites and their protective bases were incorporated into the primers (Supplementary Table S1). Then, *mepB* was amplified from genomic DNA of tested isolates by PCR. The PCR products were digested with restriction endonucleases *EcoR*I and *Xba*I, and then ligated into an expression vector pUCP24 that had been treated with the same restriction endonucleases using the T4 DNA ligase (Takara). The recombinant plasmids were transformed into *E. coli* DH5α, which were grown on Luria-Bertani agar plates supplemented with gentamicin (20 μg/ml), and then further verified by colony PCR and sequencing.

Antimicrobial susceptibility testing for the transformants was performed to verify the function of *mepB* using the broth microdilution method. The recipient *E. coli* DH5α or *E. coli* DH5α carrying the vector pUCP24 (pUCP24/DH5α) was used as the negative control. *E. coli* ATCC 25922 was used as the quality control strain.

### Effect of efflux pump inhibitors

According to past study (Zhu et al., 2021), the effect of efflux pump inhibitor carbonyl cyanide m-chlorophenylhydrazone (CCCP, 0.4 μg/ml) on the tetracyclines activity was determined by the broth microdilution method in the presence and absence of the efflux pump inhibitors. The effects of efflux pump inhibitors were interpreted as follows: it was considered as a positive efflux pump phenotype when the MIC of antibiotics decreased to 4-fold or more after the supplementation of the efflux pump inhibitors (Zhang et al., 2018).

### Antibiotic treatment and total RNA isolation

To decipher the mechanisms of resistance to tetracyclines, ten tetracyclines-resistant *S. aureus* strains with a positive efflux pump phenotype, a randomly selected equal number of tetracyclines-susceptible strains and *S. aureus* ATCC 29213 were used to measure the transcriptional levels of the efflux pump encoding-genes *mepA*, *tet38*, *tet*(K), and *tet*(L).

First, a single pure colony of *S. aureus* was randomly picked, inoculated into 3 ml of fresh LB broth, and allowed to grow to the logarithmic phase. Then, 30 μl of the overnight culture was transferred into 2.97 ml fresh LB broth without or with 1/2 × MIC (subinhibitory concentrations) of eravacycline, omadacycline, or tigecycline, respectively (Xu et al., 2020). Next, which the cells were harvested, and their total bacterial RNA was extracted by using the Bacterial RNA Miniprep Kit (Biomiga, Shanghai, China) in accordance with the manufacturer’s instructions. The extracted RNA was reverse transcribed into cDNA by using the PrimeScript RT Reagent Kit (Takara Bio Inc., Shiga, Japan).

### RT-qPCR analysis

RT-qPCR was performed on the CFX-96 TouchTM Real-Time PCR System (Bio-Rad, Hercules, CA, United States) using the TB Green Premix Ex Taq II (Tli RNase H Plus) (2×) (Takara, Japan). The internal control gene *gyrB* was used to normalize the expression of target genes, and the data were analyzed by using the 2^−ΔΔCt^ method. The relative expression of the target gene was normalized to that of *S. aureus* ATCC 29213. All RT-qPCR were performed in triplicate using 3 independent RNA samples. The RT-qPCR primers were listed in Supplementary Table S1.

### Multi-locus sequence typing

MLST was conducted by amplifying and sequencing 7 housekeeping genes (*arcC*, *aroE*, *glpF*, *gmK*, *pta*, *tpi*,and *yqiL*) using specific primers acquired form PubMLST website.^1^ The PCR products were sequenced, and the sequences were compared with those available from the MLST database^2^ to obtain the allelic numbers, STs and clonal complexes.

### Statistical analyses

Statistical analyses of the gene expression levels were performed with the GraphPad Prism 8.0 software using the paired Student’s *t*-test, with *p* < 0.05 was considered to be significant.

## Results

### Antimicrobial activity of new generation tetracyclines against clinical *Staphylococcus aureus* strains

Among 1,017 *S. aureus* isolates, three eravacycline, omadacycline, and tigecycline co-resistant, six omadacycline and tigecycline co-resistant, and 32 omadacycline-resistant strains were screened from tetracycline-resistant strains, but not from tetracycline-susceptible strains. Moreover, eravacycline (resistance rate, 0.29%) showed the lowest resistance rate when comparing the results of omadacycline (resistance rate, 4.03%) and tigecycline (resistance rate, 0.88%). As shown in Table 1, the MIC_50_ and MIC_90_ of eravacycline, omadacycline, and tigecycline against for 1,017 *S. aureus* isolates were 0.12 and 0.25 μg/ml, 0.5 and 1 μg/ml, and 0.12 and 0.25 μg/ml, respectively. In comparison, MIC_50_ and MIC_90_ values of omadacycline against *S. aureus* increased 4-fold when compared with that of eravacycline and tigecycline. Overall, these data indicated that eravacycline and tigecycline had more excellent antimicrobial activity against *S. aureus* strains than omadacycline, including tetracycline-resistant strains.

**Table 1:** Antimicrobial activity of eravacycline, omadacycline and tigecycline against *Staphylococcus aureus* isolates.

Tetracycline agents	Organism group (no. of isolates tested)	No. (cumulative %) of isolates inhibited at MIC (μg/mL) of:							MIC_50/90_ (μg/mL)	Resistance rate (%)
		≤0.06	0.12	0.25	0.5	1	2	≥4		
Eravacycline	TET-R (577)	101	289	158	26	1	1	1	0.12/0.25	0.52
	TET-S (440)	172	254	13	1	0	0	0	0.12/0.12	0
	Total (1,017)	273	543	171	27	1	1	1	0.12/0.25	0.29
Omadacycline	TET-R (577)	0	60	67	282	127	6	35	0.5/1	7.11
	TET-S (440)	13	44	147	213	23	0	0	0.5/0.5	0
	Total (1,017)	13	104	214	495	150	6	35	0.5/1	4.03
Tigecycline	TET-R (577)	61	260	183	64	6	2	1	0.12/0.5	1.56
	TET-S (440)	108	315	15	2	0	0	0	0.12/0.12	0
	Total (1,017)	169	575	198	66	6	2	1	0.12/0.25	0.88

MIC, minimum inhibitory concentration; TET-R, tetracycline-resistant; TET-S, tetracycline-susceptible.

**Figure 1:**
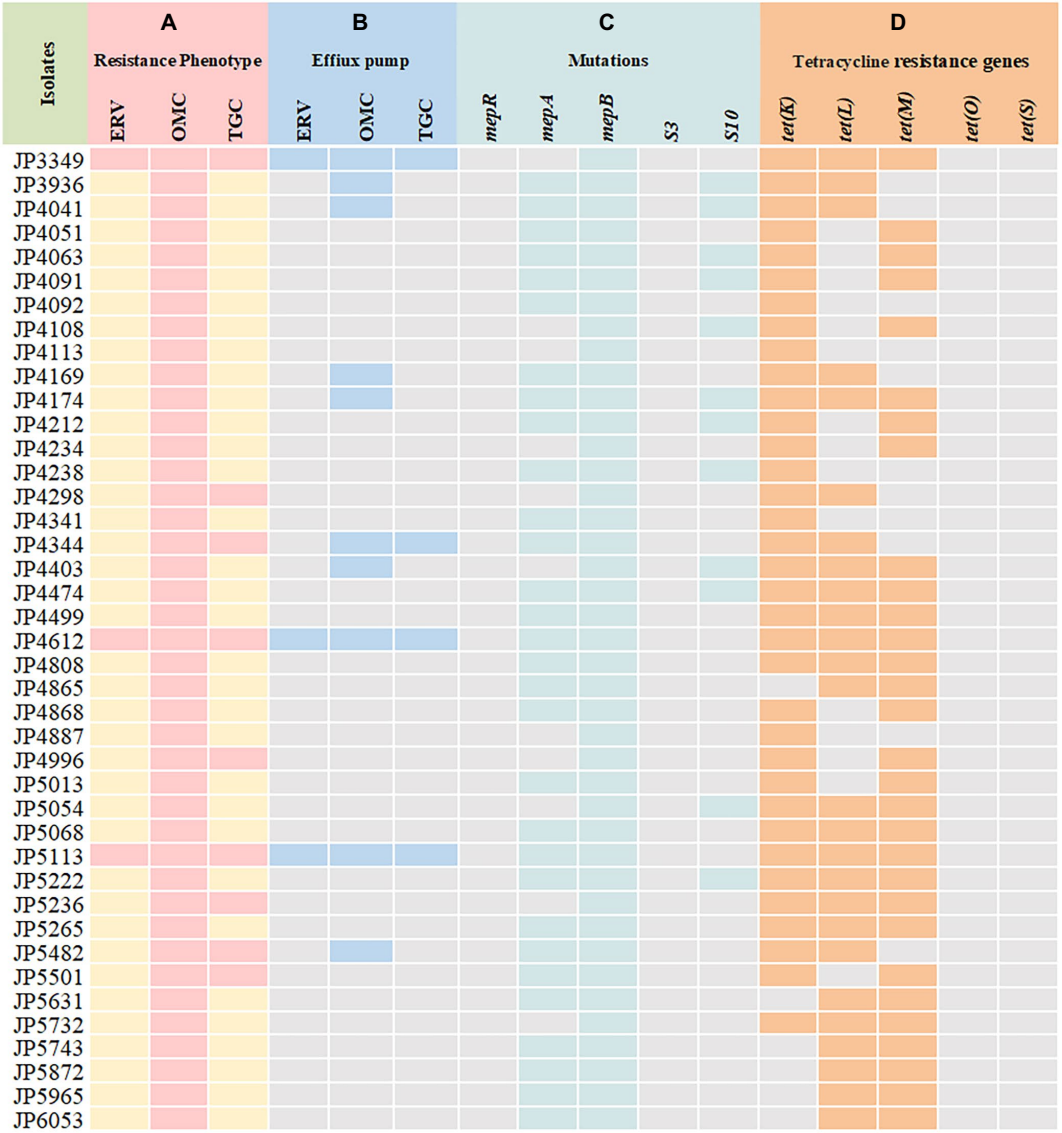
Tetracyclines resistance mechanisms determined in the clinical *Staphylococcus aureus* isolates. **(A)** Resistance phenotype of *S. aureus* to tetracyclines; **(B)** carbonyl cyanide 3-chlorophenylhydrazone (CCCP) was used to detect the activity of efflux pumps; **(C)** mutations in MepRAB efflux pump-encoding genes and 30S ribosome subunits; **(D)** PCR detection of tetracycline resistance genes. ERV, eravacycline; OMC, omadacycline; TGC, tigecycline; pink and yellow rectangles indicate resistant and susceptible phenotypes, respectively; Blue rectangles indicate strains with a positive efflux pump phenotype; Green rectangles represent genetic mutations that are only present in the resistant strains; Orange rectangles indicate strains harboring tetracycline resistance genes.

Out of the 41 tetracyclines-resistant *S. aureus* isolates, most of them possessed two or more tetracycline resistance genes: *tet*(K) (*n* = 5), *tet*(L) (*n* = 1), *tet*(K) + *tet*(L) (*n* = 5), *tet*(K) + *tet*(M) (*n* = 10), *tet*(L) + *tet*(M) (*n* = 6), *tet*(K) + *tet*(L) + *tet*(M) (*n* = 14). None *tet*(O) and *tet*(S) was detected (Table 2). Notably, three different tetracycline resistance genes [*tet*(K) + *tet*(L) + *tet*(M)] were detected simultaneously in the three tetracyclines co-resistant strains (Figure 1).

**Table 2:** Geographic distribution of 41 tetracyclines-resistant *Staphylococcus aureus* isolates.

Tetracycline resistance genes	No. of isolates	Carrier rate (%)
*tet*(S)	0	0.00
*tet*(O)	0	0.00
*tet*(K)	5	12.20
*tet*(L)	1	2.43
*tet*(K) + *tet*(L)	5	12.20
*tet*(K) + *tet*(M)	10	24.39
*tet*(L) + *tet*(M)	6	14.63
*tet*(K) + *tet*(L) + *tet*(M)	14	34.15

### Tetracyclines-resistant *Staphylococcus aureus* isolates presented a MDR phenotype

A total of 41 tetracyclines-resistant strains presented MDR phenotype to commonly used clinical antibiotics, which exhibited a high frequency of antimicrobial resistance to tetracycline, CIP, GEN, LVX, ERY, and OXA (Table 3). However, the MDR phenotype was not observed in tetracyclines-susceptible isolates.

**Table 3:** Characteristics of the resistance spectrum of tetracyclines-resistant and-susceptible *Staphylococcus aureus* isolates.

Isolates	MIC (μg/mL)											
	New tetracycline agents			Commonly used clinical antibiotics								
	ERV	OMC	TGC	TET	CIP	GEN	LVX	ERY	LNZ	OXA	RIF	VAN
JP3349	4^R^	4^R^	≥8^R^	32^R^	128^R^	128^R^	16^R^	128^R^	1	≥256^R^	2	1
JP3936	0.12	2^R^	0.25	32^R^	64^R^	128^R^	1	0.5	2	≥256^R^	2	2
JP4041	0.5	4^R^	0.5	32^R^	128^R^	128^R^	1	0.5	2	≥256^R^	8^R^	2
JP4051	0.12	2^R^	0.5	32^R^	256^R^	128^R^	0.25	1	2	≥256^R^	2	1
JP4063	0.12	2^R^	0.5	32^R^	64^R^	128^R^	0.25	1	2	≥256^R^	2	2
JP4091	0.5	4^R^	0.5	32^R^	2	128^R^	1	0.5	2	≥256^R^	4^R^	2
JP4092	0.12	4^R^	0.5	32^R^	64^R^	128^R^	0.5	0.5	2	≥256^R^	2	1
JP4108	0.5	4^R^	0.5	64^R^	64^R^	128^R^	0.25	0.5	2	≥256^R^	2	2
JP4113	0.25	4^R^	0.5	64^R^	256^R^	128^R^	0.25	1	2	≥256^R^	4^R^	2
JP4169	0.12	4^R^	0.5	64^R^	256^R^	4	32^R^	64^R^	2	≥256^R^	≤0.125	2
JP4174	0.5	4^R^	0.5	64^R^	128^R^	16^R^	16^R^	32^R^	1	≥256^R^	≤0.125	1
JP4212	0.12	8^R^	0.5	64^R^	128^R^	0.5	32^R^	64^R^	1	≥256^R^	≤0.125	2
JP4234	0.5	8^R^	0.5	64^R^	256^R^	4	32^R^	128^R^	1	≥256^R^	≤0.125	2
JP4238	0.5	4^R^	0.5	32^R^	256^R^	128^R^	32^R^	128^R^	2	≥256^R^	≤0.125	1
JP4298	0.5	4^R^	1^R^	64^R^	128^R^	128^R^	16^R^	64^R^	1	≥256^R^	≤0.125	1
JP4341	0.5	4^R^	0.5	32^R^	64^R^	128^R^	16^R^	64^R^	1	≥256^R^	≤0.125	1
JP4344	0.5	8^R^	1^R^	32^R^	256^R^	16^R^	16^R^	≥256^R^	2	≥256^R^	≤0.125	1
JP4403	0.12	2^R^	0.5	32^R^	256^R^	32^R^	16^R^	32^R^	2	≥256^R^	≤0.125	2
JP4474	0.5	4^R^	0.5	32^R^	128^R^	128^R^	16^R^	64^R^	2	≥256^R^	≤0.125	2
JP4499	0.5	4^R^	0.5	16^R^	256^R^	128^R^	16^R^	32^R^	1	≥256^R^	≤0.125	1
JP4612	2^R^	4^R^	2^R^	64^R^	64^R^	≥256^R^	256	≥256^R^	1	≥256^R^	≤0.125	2
JP4808	0.5	8^R^	0.5	32^R^	128^R^	128^R^	16^R^	32^R^	1	≥256^R^	≤0.125	1
JP4865	0.5	4^R^	0.5	32^R^	128^R^	128^R^	16^R^	64^R^	1	≥256^R^	≤0.125	2
JP4868	0.5	4^R^	0.5	32^R^	256^R^	128^R^	16^R^	64^R^	2	≥256^R^	≤0.125	1
JP4887	0.5	4^R^	0.5	64^R^	128^R^	128^R^	16^R^	128^R^	1	≥256^R^	≤0.125	2
JP4996	0.5	4^R^	1^R^	32^R^	64^R^	128^R^	32^R^	64^R^	1	≥256^R^	≤0.125	1
JP5013	0.12	2^R^	0.25	32^R^	128^R^	128^R^	16^R^	64^R^	1	≥256^R^	≤0.125	1
JP5054	0.5	4^R^	0.5	64^R^	256^R^	128^R^	16^R^	32^R^	1	≥256^R^	≤0.125	1
JP5068	0.5	4^R^	0.5	32^R^	256^R^	0.5	16^R^	32^R^	1	≥256^R^	≤0.125	1
JP5113	1^R^	8^R^	1^R^	32^R^	128^R^	≥256^R^	16^R^	32^R^	1	≥256^R^	≤0.125	1
JP5222	0.5	4^R^	0.5	32^R^	256^R^	128^R^	32^R^	32^R^	1	≥256^R^	≤0.125	2
JP5236	0.5	4^R^	1^R^	64^R^	128^R^	128^R^	16^R^	128^R^	2	≥256^R^	≤0.125	1
JP5265	0.12	4^R^	0.5	32^R^	256^R^	128^R^	16^R^	64^R^	1	≥256^R^	≤0.125	2
JP5482	0.12	4^R^	1^R^	64^R^	256^R^	128^R^	16^R^	32^R^	2	≥256^R^	≤0.125	1
JP5501	0.5	8^R^	1^R^	64^R^	128^R^	128^R^	16^R^	64^R^	2	≥256^R^	≤0.125	1
JP5631	0.5	4^R^	0.5	16^R^	128^R^	128^R^	16^R^	64^R^	2	≥256^R^	≤0.125	1
JP5732	0.5	8^R^	0.5	64^R^	128^R^	128^R^	16^R^	128^R^	1	≥256^R^	≤0.125	2
JP5743	0.5	4^R^	0.5	32^R^	256^R^	128^R^	16^R^	64^R^	1	≥256^R^	≤0.125	2
JP5872	0.5	4^R^	0.5	32^R^	256^R^	128^R^	32^R^	32^R^	2	≥256^R^	≤0.125	1
JP5965	0.5	4^R^	0.5	32^R^	256^R^	128^R^	16^R^	32^R^	1	≥256^R^	≤0.125	1
JP6053	0.12	2^R^	0.5	32^R^	256^R^	128^R^	16^R^	128^R^	2	≥256^R^	≤0.125	2
JP3694	0.12	0.5	0.25	16^R^	0.25	0.25	0.25	0.5	1	0.5	≤0.125	2
JP3798	0.12	0.5	0.12	32^R^	0.5	128^R^	0.25	0.5	2	0.5	≤0.125	0.5
JP4164	0.12	0.5	0.25	16^R^	0.5	0.5	0.5	≥256^R^	1	≥256^R^	≤0.125	0.5
JP4200	0.06	0.5	0.12	32^R^	0.5	0.25	0.5	≥256^R^	2	≥256^R^	≤0.125	1
JP4210	0.12	0.5	0.25	16^R^	2	0.25	1	≥256^R^	2	≥256^R^	≤0.125	1
JP4218	0.06	0.5	0.12	32^R^	0.5	0.5	0.5	≥256^R^	1	≥256^R^	≤0.125	2
JP4389	0.06	0.5	0.12	16^R^	0.5	0.25	0.25	≥256^R^	1	≥256^R^	≤0.125	0.5
JP4697	0.06	0.5	0.12	64^R^	0.25	0.25	0.25	≥256^R^	1	≥256^R^	≤0.125	1
JP5736	0.12	0.25	0.12	16^R^	0.5	0.25	0.5	1	1	≥256^R^	≤0.125	1
JP6116	0.12	0.5	0.25	16^R^	256^R^	0.5	32^R^	0.5	1	0.5	≤0.125	1

MIC, minimum inhibitory concentration; ERV, eravacycline; OMC, omadacycline; TGC, tigecycline; TET, tetracycline; CIP, ciprofloxacin; GEN, gentamicin; LVX, levofloxacin; ERY, erythromycin; LNZ, linezolid; OXA, oxacillin; RIF, rifampicin; VAN, vancomycin; Superscript “R” indicates resistance.

### Mutations in the MepRAB efflux pump and 30S ribosome subunits encoding genes

To elucidate the resistance mechanisms of *S. aureus* strains to tetracyclines, genetic mutations of the MepRAB efflux pump and 30S ribosome subunits encoding genes were determined by PCR and sequenced (Table 4; Figure 1). Compared with tetracyclines-susceptible strains and *S. aureus* ATCC 29213, the amino acid mutations L155F and T411A in *mepA* and Y58D in *S10* were frequently found in tetracyclines-resistant strains. Moreover, tetracyclines-resistant *S. aureus* isolates also contained premature stop codon of *mepA* (E130*), *mepB* (Q139*), and *S10* (K3* and Y13*). Interestingly, except for JP3349, almost all tetracyclines-resistant *S. aureus* isolates contained nucleotide deletion in *mepB* (F144fs) that translated Phe at 144 to Leu. Nevertheless, a frameshift mutation in *mepB* was not found among ten tetracyclines-susceptible *S. aureus* isolates. Therefore, we hypothesized that a frameshift mutation in *mepB* may be associated with tetracyclines resistance. In addition, *mepB* (Q139*), *mepA* (K2Q) *+ mepB* (F144fs), and *mepA* (L155F) *+ mepB* (F144fs) were detected among three tetracyclines co-resistant strains JP3349, JP4612, and JP5113, respectively. No specific mutations were detected in *mepR* and *S3* genes for tetracyclines-resistant *S. aureus* isolates compared with the tetracyclines-susceptible *S. aureus* isolates.

**Table 4:** Multi-locus sequence typing and the resistance mechanisms in tetracyclines-resistant and-susceptible *Staphylococcus aureus.*

Isolates	Type	MLST		Resistance mechanisms				
				Mutation of MepRAB efflux pump			Mutation of 30S ribosome protein	
		STs	CCs	*mepR*	*mepA*	*mepB*	*S3*	*S10*
JP3349^a^	R	ST239	CC8	None^c^	None	Q139* ^d^	None	None
JP3936	R	ST5	CC5	None	**L155F**^e^, V167I, S332I	**F144fs** ^f^	None	M1L, K3*
JP4041	R	ST5	CC5	None	E130*	**F144fs**	None	**Y58D**
JP4051	R	ST5	CC5	None	**L155F**, V167I, S332I	**F144fs**	None	K57M
JP4063	R	ST5	CC5	None	**L155F**, V167I, S332I	**F144fs**	None	**Y58D**
JP4091	R	ST5	CC5	None	V167I, S332I,**T411A**	**F144fs**	None	**Y58D**
JP4092	R	ST5	CC5	None	**L155F**, V167I, S332I	**F144fs**	None	K57M
JP4108	R	ST764	CC5	None	V167I, S332I	**F144fs**	None	**Y58D**
JP4113	R	ST764	CC5	None	V167I, S332I	**F144fs**	None	K57M
JP4169	R	ST5	CC5	None	**L155F**, V167I, S332I	**F144fs**	None	K57M
JP4174	R	ST5	CC5	None	**L155F**, V167I, S332I	**F144fs**	None	**Q4K, I6fs**
JP4212	R	ST5	CC5	None	V167I, S332I,**T411A**	**F144fs**	None	**Y58D**
JP4234	R	ST764	CC5	None	V167I, S332I	**F144fs**	None	K57M
JP4238	R	ST5	CC5	None	E130*	**F144fs**	None	M1L, K3*
JP4298^b^	R	ST764	CC5	None	None	**F144fs**	None	K57M
JP4341	R	ST5	CC5	None	E130*	**F144fs**	None	K57M
JP4344^b^	R	ST5	CC5	None	**L155F**, V167I, S332I	**F144fs**	None	K57M
JP4403	R	ST5	CC5	None	**L155F**, V167I, S332I	**F144fs**	None	**Y58D**
JP4474	R	ST5	CC5	None	E130*	**F144fs**	None	**Y58D**
JP4499	R	ST5	CC5	None	E130*	**F144fs**	None	K57M
JP4612^a^	R	ST764	CC5	None	**K2Q**, V167I, S332I	**F144fs**	None	K57M
JP4808	R	ST5	CC5	None	V167I, S332I,**T411A**	**F144fs**	None	K57M
JP4865	R	ST5	CC5	None	V167I, S332I,**T411A**	**F144fs**	None	K57M
JP4868	R	ST5	CC5	None	V167I, S332I,**T411A**	**F144fs**	None	K57M
JP4887	R	ST764	CC5	None	V167I, S332I,	**F144fs**	None	K57M
JP4996^b^	R	New1^g^	ND^h^	None	None	**F144fs**	None	K57M
JP5013	R	ST5	CC5	None	**L155F**, V167I, S332I	**F144fs**	None	K57M
JP5054	R	ST764	CC5	None	V167I, S332I	**F144fs**	None	**Y58D**
JP5068	R	ST5	CC5	None	E130*	**F144fs**	None	K57M
JP5113^a^	R	ST5	CC5	None	**L155F**, V167I, S332I	**F144fs**	None	K57M
JP5222	R	ST5	CC5	None	V167I, S332I,**T411A**	**F144fs**	None	**Y58D**
JP5236^b^	R	ST764	CC5	None	V167I, S332I	**F144fs**	None	K57M
JP5265	R	ST5	CC5	None	V167I, S332I,**T411A**	**F144fs**	None	K57M
JP5482^b^	R	ST5	CC5	None	V167I, S332I,**T411A**	**F144fs**	None	K57M
JP5501^b^	R	ST5	CC5	None	V167I, S332I,**T411A**	**F144fs**	None	K57M
JP5631	R	ST5	CC5	None	V167I, S332I,**T411A**	**F144fs**	None	K57M
JP5732	R	ST764	CC5	None	V167I, S332I	**F144fs**	None	K57M
JP5743	R	ST5	CC5	None	V167I, S332I,**T411A**	**F144fs**	None	K57M
JP5872	R	ST5	CC5	None	V167I, S332I,**T411A**	**F144fs**	None	K57M
JP5965	R	ST5	CC5	None	V167I, S332I,**T411A**	**F144fs**	None	K57M
JP6053	R	ST5	CC5	None	V167I, S332I,**T411A**	**F144fs**	None	K57M
JP3694	S	ST338	CC59	M70I	K93E, T114A, V167I, A307S S332I, A364T	T131R, G133E, K137N	None	K57M
JP3798	S	ST398	CC398	D40G, K74R	V167I, S332I	None	None	None
JP4164	S	ST59	CC59	M70I	V167I, S332I	T131R, G133E, K137N	None	None
JP4200	S	New2	ND	None	V167I, S332I	None	None	None
JP4210	S	ST59	CC59	M70I	K93E, T114A, A307S, A364T	T131R, G133E, K137N	None	None
JP4218	S	New2	ND	None	V167I, S332I	None	None	None
JP4389	S	ST59	CC59	M70I	K93E, T114A, A307S, A364T	T131R, G133E, K137N	None	None
JP4697	S	ST59	CC59	M70I	K93E, T114A, A307S, A364T	T131R, G133E, K137N	None	None
JP5736	S	New3	ND	None	I214V, V167I, S332I, A350D	None	None	None
JP6116	S	ST398	CC398	D40G, K74R	T114A, V167I, S332I, A307S A364T	None	None	None

R, tetracyclines-resistant (eravacycline/omadacycline/tigecycline-resistant) strain; S, tetracyclines-susceptible (eravacycline/omadacycline/tigecycline-susceptible) strain; MLST, multi-locus sequence typing; STs, sequence types; CCs, clonal complexes; ^a^means the co-resistant strain of eravacycline, omadacycline and tigecycline; ^b^means the co-resistant strain of omadacycline and tigecycline; None^c^ means no specific mutation; Q139*^d^ means premature stop codon at Gln139; L155F^e^ means amino acid substituted at Leu-155; F144fs^f^ means frameshift mutation in Phe at 144; New1^g^-New3 means 3 different new ST; ND^h^ means not detected. The bold font indicates that the mutation is only present in resistant strains.

### A frameshift mutation in *mepB* contributed to reduced OMC susceptibility

To verify our hypothesis, *mepB* with and without frameshift mutation was successfully cloned into *E. coli* DH5α. The MIC of tetracyclines against the recombinant strains is shown in Table 5. Compared with negative control strain pUCP24/DH5α, pUCP24-*mepB*/DH5α-JP3936 and pUCP24-*mepB*/DH5α-JP4612 with a frameshift mutation in *mepB* showed increased MIC levels against omadacycline (MIC increased from 0.5–2 μg/ml, 4-fold), but not in pUCP24-*mepB*/DH5α-JP4200 without frameshift mutation in *mepB*. Besides, the MICs of eravacycline and tigecycline did not change compared with that of pUCP24/DH5α among these recombinant strains, regardless of whether the *mepB* they carried underwent frameshift mutation. These data suggested that a frameshift mutation in *mepB* contributed to reduce omadacycline susceptibility.

**Table 5:** Changes in the MIC of tetracyclines and the information of isolates used in the cloning experiments in this study.

Isolates	MIC (μg/mL)			Description
	ERV	OMC	TGC	
JP3936	0.12	2	0.25	OMC-R and ERV/TGC-S strain, with frameshift mutation of *mepB*
JP4612	2	4	2	Tigecyclines-resistant, with frameshift mutation in *mepB*
JP4200	0.06	0.5	0.12	Tigecyclines-susceptible-S, without frameshift mutation in *mepB*
*E. coli* ATCC 25922	0.12	1	0.12	Used as a control strain
*E. coli* DH5α	0.06	0.5	0.12	Used as a host for the PCR product clone
pUCP24/DH5α	0.06	0.5	0.12	*E. coli* DH5α carrying cloning expression vector pUCP24
pUCP24-*mepB*/DH5α-JP3936	0.06	2	0.12	*mepB* of JP3936 was cloned into the expression vector pUCP24 and transformed into *E. coli* DH5 α
pUCP24-*mepB*/DH5α-JP4612	0.06	2	0.12	*mepB* of JP4612 was cloned into the expression vector pUCP24 and transformed into *E. coli* DH5 α
pUCP24-*mepB*/DH5α-JP4200	0.06	0.5	0.12	*mepB* of JP4200 was cloned into the expression vector pUCP24 and transformed into *E. coli* DH5 α

MIC, minimum inhibitory concentrations; ERV, eravacycline; OMC, omadacycline; TGC, tigecycline; OMC-R, omadacycline-resistant; ERV/TGC-S, eravacycline- and tigecycline-susceptible; Tetracyclines-resistant (eravacycline/omadacycline/tigecycline-resistant); Tetracyclines-susceptible (eravacycline/omadacycline/tigecycline-susceptible).

### Efflux pump phenotype assay

The effect of efflux pump inhibitor on the MIC of tetracycline agents was evaluated. As shown in Figure 1 and Supplementary Table S2, among 41 tetracyclines-resistant strains, ten omadacycline-resistant, three eravacycline-resistant and four tigecycline-resistant strains showed positive efflux pump phenotype, including. These results suggested the possible effect of efflux pumps on the occurrence of tetracyclines resistance. Therefore, these strains with a positive efflux pump phenotype were further selected to detect of the expression of efflux pump-encoding genes.

### Expression analysis of efflux pump-encoding genes

**Figure 2:**
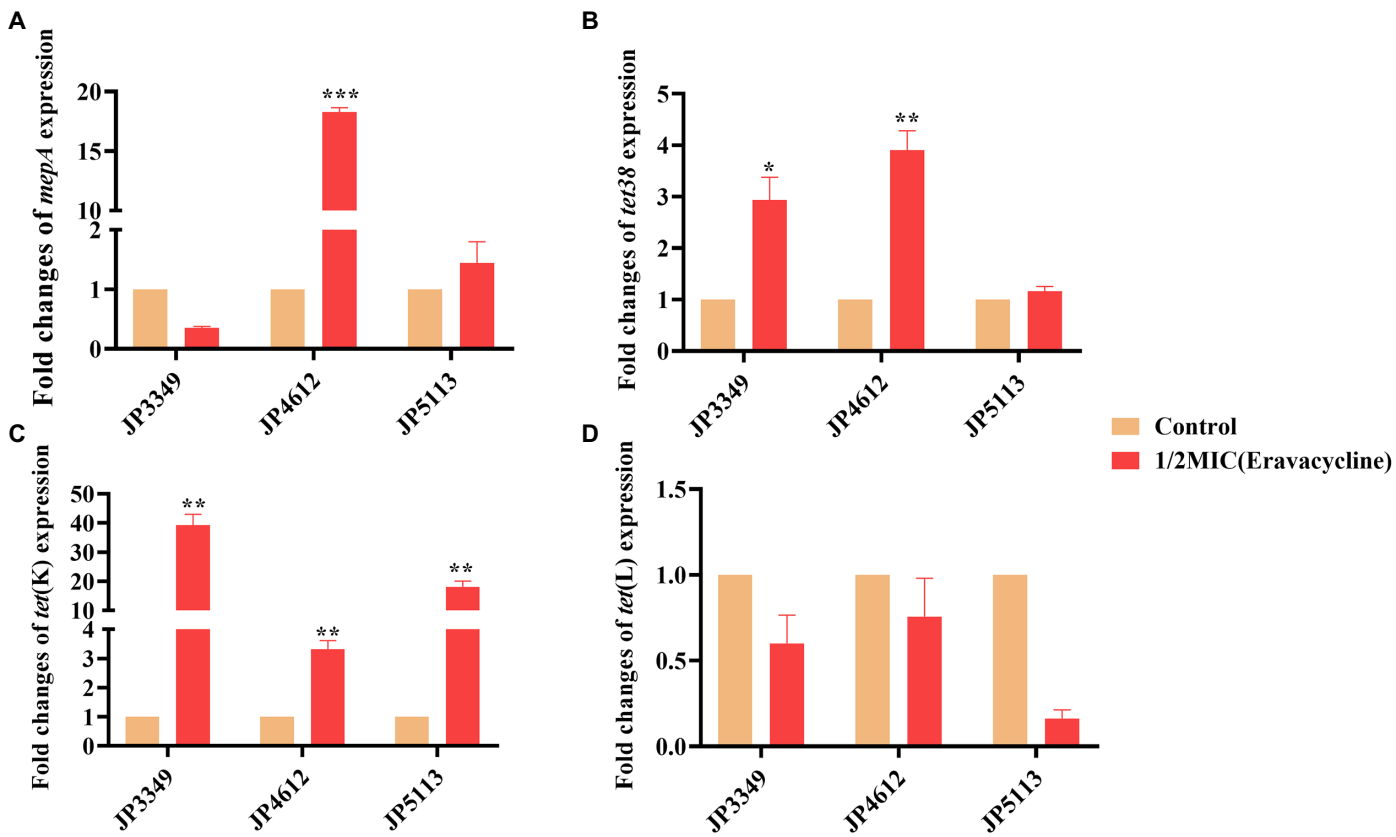
Fold changes in the expression of efflux pump-encoding genes in eravacycline-resistant *Staphylococcus aureus* exposed to 1/2 × MIC eravacycline concentrations. **(A–D)** The expression levels of *mepA*, *tet38*, *tet*(K), and *tet*(L) genes. The values were normalized based on the internal control gene, *gyrB*. Data from the resistant strain with untreated 1/2 × MIC eravacycline were normalized to 1 to allow the comparison of data across different samples. Data represented the mean values from 3 independent experiments with error bars indicating standard deviations, and asterisks denoted the significance of differences in the expression by paired Student’s *t*-test (**p < 0.01; ***p < 0.001).

**Figure 3:**
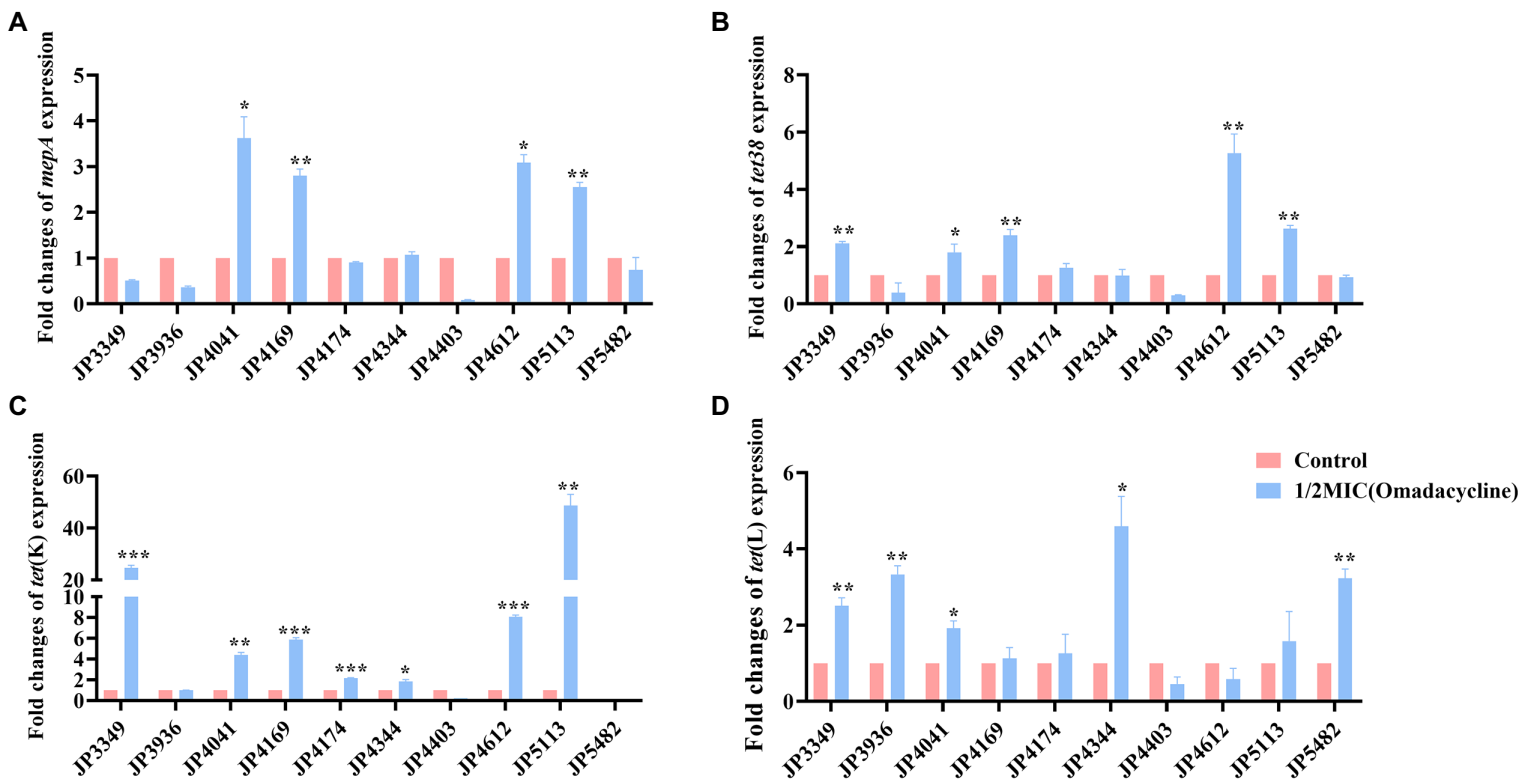
Fold changes in the expression of efflux pump-encoding genes in omadacycline-resistant *Staphylococcus aureus* exposed to 1/2 × MIC omadacycline concentrations. **(A–D)** The expression levels of *mepA*, *tet38*, *tet*(K), and *tet*(L) genes. The values were normalized based on the internal control gene, *gyrB*. Data from the resistant strain with untreated 1/2 × MIC omadacycline were normalized to 1 to allow the comparison of data across different samples. Data represented the mean values from 3 independent experiments with error bars indicating standard deviations, and asterisks denoted the significance of differences in the expression by paired Student’s *t*-test (*p < 0.05; **p < 0.01; ***p < 0.001).

**Figure 4:**
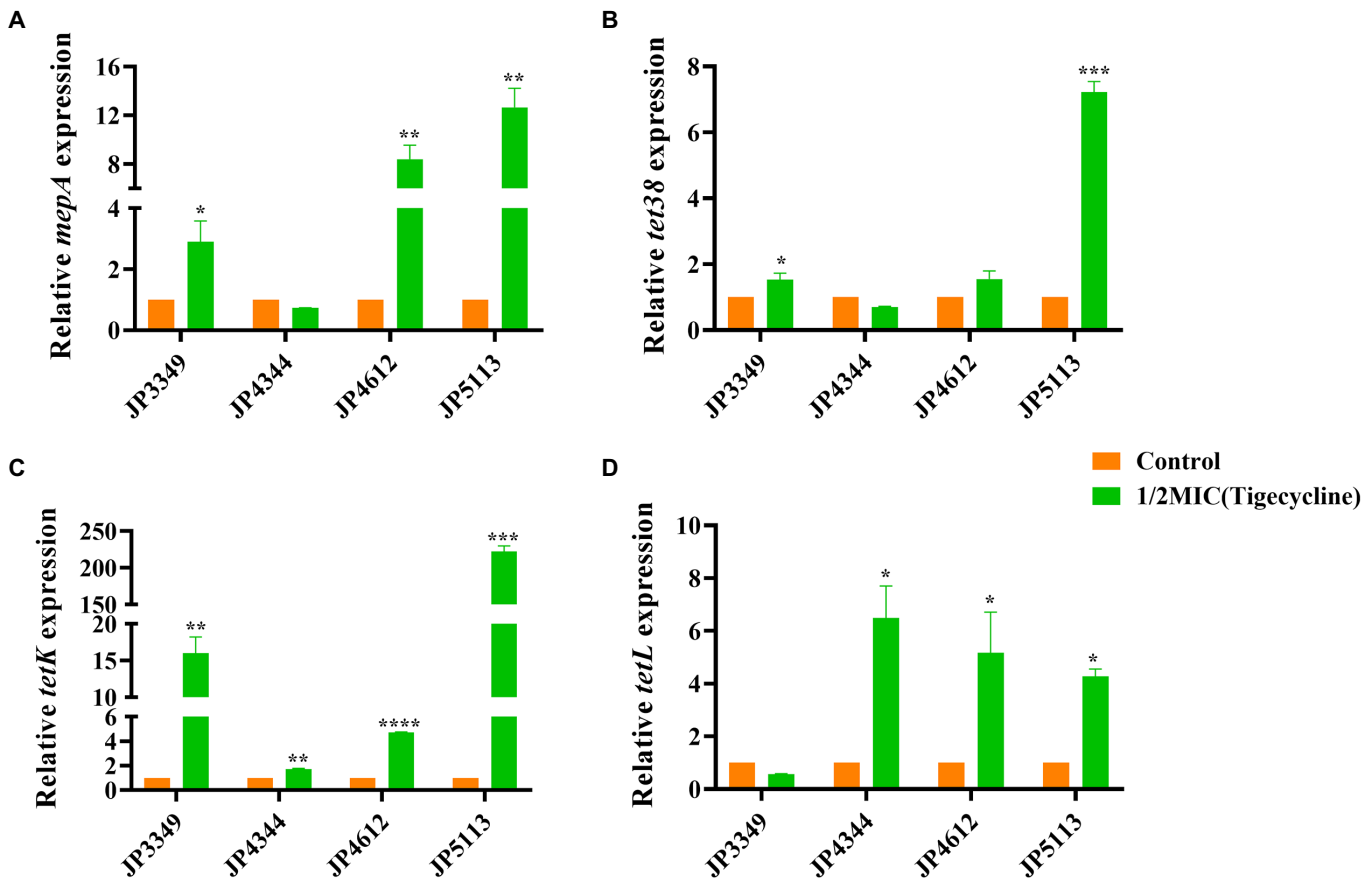
Fold changes in the expression of efflux pump-encoding genes in tigecycline-resistant *Staphylococcus aureus* exposed to 1/2 × MIC tigecycline concentrations. **(A–D)** The expression levels of *mepA*, *tet38*, *tet*(K), and *tet*(L) genes. The values were normalized based on the internal control gene, *gyrB*. Data from the resistant strain with untreated 1/2 × MIC tigecycline were normalized to 1 to allow the comparison of data across different samples. Data represented the mean values from 3 independent experiments with error bars indicating standard deviations, and asterisks denoted the significance of differences in the expression by the paired Student’s *t*-test (*p < 0.05; **p < 0.01; ***p < 0.001; ****p < 0.0001).

The transcriptional levels of the efflux pump-encoding genes *mepA*, *tet38*, *tet*(K), and *tet*(L) were determined in the presence of the tetracycline agents with 1/2 × MIC concentrations (Supplementary Table S3). The expression of *mepA*, *tet38*, and *tet*(K) increased significantly in the three eravacycline-resistant strains treated with 1/2 × MIC eravacycline than in the control strains (0 MIC; Figure 2). An increased expression of efflux pump-encoding genes was observed in different omadacycline-resistant strains after exposure to 1/2 × MIC omadacycline (Figure 3). Furthermore, the expression of *mepA*, *tet38*, *tet*(K), and *tet*(L) was upregulated in the four tigecycline-resistant strains after stimulation with 1/2 × MIC tigecycline, (Figure 4). To summarize, different efflux pump genes were upregulated to varying degrees after the resistant strains were exposed to 1/2 × MIC tetracycline agents.

### Relationship of STs with tetracyclines susceptibility

**Figure 5:**
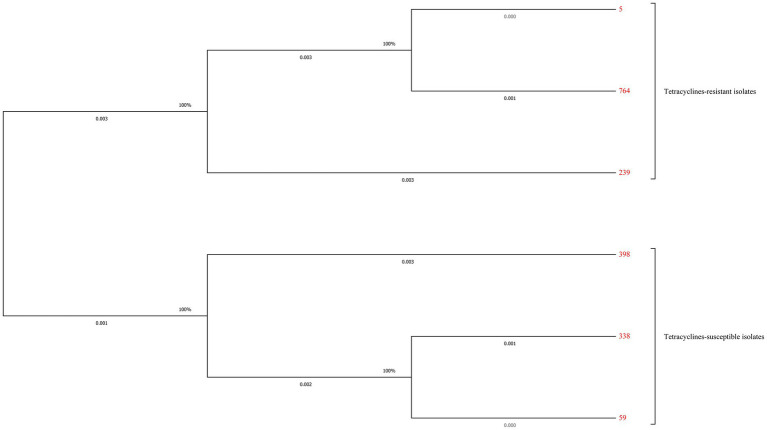
Maximum-likelihood trees of 41 tetracyclines-resistant and 10 tetracyclines-susceptible *Staphylococcus aureus* isolates based on the concatenated sequences of MLST. Tetracyclines-resistant and-susceptible isolates were divided into two major branches. ST5 (*n* = 30), ST764 (*n* = 9), and ST239 (*n* = 1) were seen in the tetracyclines-resistant isolates, and ST59 (*n* = 4), ST398 (*n* = 2), and ST338 (*n* = 1) were observed in tetracyclines-susceptible isolates.

Totally six STs and three different new STs were found by the MLST analysis in the tested *S. aureus* isolates. The distribution of these STs between the tetracyclines-resistant and-susceptible *S. aureus* was not the same. As shown in Table 4, MLST results showed that one tetracyclines-resistant and three-susceptible strains belonged to the new STs. The phylogenetic tree showed tetracyclines-resistant and-susceptible isolates were divided into two major branches. ST5 (*n* = 30), ST764 (*n* = 9), and ST239 (*n* = 1) were seen in the tetracyclines-resistant isolates, and ST59 (*n* = 4), ST398 (*n* = 2), and ST338 (*n* = 1) were observed in tetracyclines-susceptible isolates (Figure 5; Supplementary Figure S1).

## Discussion

Studies have shown that eravacycline, omadacycline, and tigecycline are broad-spectrum antibiotics, which act on several Gram-positive and-negative organisms (Zhanel et al., 2018; Dowzicky and Chmelařová, 2019; Xiao et al., 2020). Consistent with previously published studies (Li et al., 2020; Xiao et al., 2020), an excellent antimicrobial activity of these tetracycline agents was observed against the 1,017 *S. aureus* isolates. In the present study, we compared the antimicrobial effects of them, the results showed that eravacycline and tigecycline, especially eravacycline, had more power antimicrobial effect than omadacycline against tested *S. aureus*. Furthermore, out of the 1,017 *S. aureus*, we successfully screened 41 tetracyclines-resistant isolates. Three tetracyclines co-resistant strains were observed among them, revealing a cross-resistance toward eravacycline, omadacycline, and tigecycline in the clinical *S. aureus* isolates, which was also observed in *Streptococcus agalactiae* (Li et al., 2020; Li G. et al., 2022). Hence, it is important to monitor and detect the resistance of tetracyclines, in order to prevent their cross-resistance leading to treatment failure. We will focus on the cross-resistance mechanism of these new generation tetracycline agents in future studies.

New generation tetracycline agents can overcome the resistance mechanisms of tetracycline and also exhibit antibacterial activity against strains containing tetracycline resistance genes, including *tet*(K), *tet*(L), *tet*(M), *tet*(O), and *tet*(S) (Nguyen et al., 2014; Linkevicius et al., 2016; Zhanel et al., 2018). In this study, eravacycline and tigecycline exhibited great antibacterial activity against clinical *S. aureus* isolates harboring the tetracycline resistance genes. However, eravacycline, omadacycline, or tigecycline were not able to against the isolates (JP3349, JP4612 and JP5113) harboring *tet*(K) + *tet*(L) + *tet*(M). The finding implies that the coexistence of multiple tetracycline resistance genes may be related to the resistance to new generation tetracycline agents in *S. aureus* isolates, which is similar to the findings of Boukthir *et al*. in enterococci (Boukthir et al., 2020). Furthermore, among the 41 tetracyclines-resistant isolates, strains harboring *tet*(K) alone or in combination with other tetracycline resistance genes were predominant. Therefore, the effect of *tet*(K) on tetracyclines susceptibility needs further studied. Consistent with previous studies (Zhang et al., 2018; Bai et al., 2019), *tet*(O) and *tet*(S) were not detected in the tested strains and need further investigation in other *S. aureus* isolates.

The analysis of 41 tetracyclines-resistant isolates showed that the accumulation of several resistance mechanisms resulted in tetracyclines resistance. Accumulating evidence showed that amino acid substitutions at S10 might be associated with reduced tetracyclines susceptibility (Beabout et al., 2015; Bai et al., 2019; Wang et al., 2020). We also detected different mutation sites (such as M1L and Y58D) in S10 in the tetracyclines-resistant isolates. K57M was found in both tetracyclines-resistant and-susceptible strains, suggesting that it was not associated with tetracyclines resistance (Fang et al., 2020). Consistent with the findings of Wang *et al*. (Wang et al., 2020), no mutations were detected in the 30S ribosome protein S3, illustrating that S3 was not the predominant factor contributing to tetracyclines resistance in *S. aureus*.

Another important resistance mechanism is the active efflux pump. The MepRAB efflux pump, a novel MATE family efflux pump, plays a key role in tigecycline resistance in *S. aureus* (McAleese et al., 2005; Fang et al., 2020), however, whether it affects eravacycline and omadacycline resistance is unclear. We first analyzed ta mutation in MepRAB efflux pump-encoding genes. The PCR results showed amino acid substitution mutations and premature termination in *mepA* were found, which might be correlated to tetracyclines resistance. In previous study, *mepR* mutations have already been related to tigecycline resistance in *S. aureus* isolates (Dabul et al., 2018). Conversely, we did not detect mutations in *mepR*, suggesting that it might not affect tetracyclines susceptibility in these tested isolates. Interestingly, the frameshift mutation in *mepB* was observed frequently in tetracyclines-resistant strains but not in tetracyclines-susceptible strains. Cloning experiment further confirmed that a frameshift mutation in *mepB* might contribute to omadacycline resistance in *S. aureus*. MepB has been considered a hypothetical protein with unknown functions until a study by Agah *et al*. revealed that *mepB* played a role in responding to antimicrobials by interacting with nucleic acids (McAleese et al., 2005; Agah et al., 2014). To the best of our knowledge, this is the first study to demonstrate that a frameshift mutation in *mepB* might mediate omadacycline resistance in *S. aureus*. Second, we also comprehensively analyzed the levels of the MepRAB efflux pump in the tetracyclines-resistant strains. We observed that the overexpression of MepRAB efflux pump-encoding genes affected tigecycline, eravacycline and omadacycline resistance.

The isolates were exposure in 1/2 × MIC tetracycline agents to understand the role of different efflux pumps. We found that, different efflux pump-encoding genes, especially *tet*(K), upregulated to varying degrees after the resistant strains were stimulated by 1/2 × MIC tetracyclines, suggesting that the active *tet*(K) efflux pump was primarily responsible for tetracyclines resistance (Bai et al., 2019; Wang et al., 2020). Although previous study showed that tnovel *tet*(L) efflux pump variants affected the susceptibility of eravacycline and tigecycline (Wang et al., 2021), Wang *et al.* and the present study showed that the *tet*(L) overexpression might not confer resistance to eravacycline (Wang et al., 2020), however, it might confer resistance to omadacycline and tigecycline in *S. aureus*. Besides conferring resistance to tetracycline (Chen and Hooper, 2018), our study revealed that the active *tet38* efflux pump might be associated with resistance to eravacycline, tigecycline and omadacycline.

The MLST analyses showed that the most tetracyclines-resistant *S. aureus* belonged to ST5, a globally disseminated and highly pathogenic lineage (Hau et al., 2018), and ST764, a variant of the ST5 lineage.

Combined with drug sensitivity, we found that the ST5 and ST764 clonotype isolates were MDR strains. Previous studies have reported that ST5 and ST764 MRSA strains were hypervirulent and MDR, and dominated *S. aureus* infections in China (Takano et al., 2013; Jian et al., 2021). For the first time, our findings revealed that ST5 and its variant ST764 dominated tetracyclines-resistant *S. aureus* strains, suggesting that tetracyclines-resistant *S. aureus* strains might pose a serious clinical threat hence more attention should be paid to their prevention and control.

However, our work also has some limitations. Tetracyclines-resistant *S. aureus* strains were only collected from the same hospital, which might lead to deviation in our results. It is necessary to use other pathogen to verify our findings, as the efficacy and resistance mechanisms of tetracyclines and distribution of ST type might be different in different hospital. Besides, the cross-resistance mechanism of these new generation tetracyclines was not illustrated in this work. Thus, the resistance and cross-resistance mechanisms of these new generation tetracyclines should be further investigated.

## Conclusion

To conclude, eravacycline and tigecycline showed more excellent antibacterial activity against *S. aureus*, including tetracycline-resistant isolates, than omadacycline did. Tetracyclines resistance resulted from an accumulation of several resistance mechanisms. The coexistence of multiple tetracycline resistance genes may contribute to the emergence of tetracyclines resistance. Moreover, mutations in *mepA* and *S10* might play crucial role in tetracyclines resistance. Importantly, we reported that frameshift mutations in *mepB* contributed to reduced omadacycline susceptibility. Furthermore, *mepA*, *tet38*, *tet*(K) and *tet*(L) overexpression reduced tetracyclines susceptibility. Moreover, a major clonal dissemination type, ST5, and its variant ST764 were determined in most tetracycline-resistant strains, suggesting that these strains might possess the risk of clonal transmission and require further investigation.

## Data availability statement

The original contributions presented in the study are included in the article/Supplementary material, further inquiries can be directed to the corresponding authors.

## Author contributions

WZ participated in the design of the study, carried out the experiments, analyzed the data, and drafted the manuscript. XZ, YL, and YZ participated in experiments and data analyses. MX and SW provided the analysis of the results. YS participated in analysis and discussions. LC and TZ designed the study, participated in data analyses, and provided critical revisions of the manuscript. All authors contributed to the revision of the manuscript and approved the final version for submission.

## Conflict of interest

The authors declare that the research was conducted in the absence of any commercial or financial relationships that could be construed as a potential conflict of interest.

## Publisher’s note

All claims expressed in this article are solely those of the authors and do not necessarily represent those of their affiliated organizations, or those of the publisher, the editors and the reviewers. Any product that may be evaluated in this article, or claim that may be made by its manufacturer, is not guaranteed or endorsed by the publisher.

## Supplementary material

The Supplementary material for this article can be found online at: https://www.frontiersin.org/articles/10.3389/fmicb.2022.1043736/full#supplementary-material

## Footnotes

^1^ http://pubmlst.org/organisms/staphylococcus-aureus/primers

^2^ http://saureus.mlst.net/
