# Supplementary material for: In vitro antimicrobial activity and resistance mechanisms of the new generation tetracycline agents, eravacycline, omadacycline, and tigecycline against clinical Staphylococcus aureus isolates
Source: Front Microbiol. 2022 Nov 22;13:1043736. doi: 10.3389/fmicb.2022.1043736 (PMC9722764; doi:10.3389/fmicb.2022.1043736)
Supplement: Supplementary file 1 [file Data_Sheet_1.docx]

Supplementary Material

# Supplementary Figures and Tables

## Supplementary Tables

**Table S1 Primers used in this study**

| Target gene | Primer | Primer sequence (5'-3') | Amplicon size (bp) | Reference |
| --- | --- | --- | --- | --- |
| Primers used for PCR | | | | |
| *tet*(K) | *tet*(K)-F | TCGATAGGAACAGCAGTA | 139 | (Bai et al., 2019) |
|  | *tet*(K)-R | CAGCAGATCCTACTCCTT |  |  |
| *tet*(L) | *tet*(L)-F | GTAACCAGCCAACTAATGAC | 908 | (Bai et al., 2019) |
|  | *tet*(L)-R | TTGGATCGATAGTAGCC |  |  |
| *tet*(M) | *tet*(M)-F | CAATACAATAGGAGCAAGC | 974 | (Bai et al., 2019) |
|  | *tet*(M)-R | CGAACAAGAGGAAAGCATAAG |  |  |
| *tet*(O) | *tet*(O)-F | AACTTAGGCATTCTGGCTCAC | 515 | (Bai et al., 2019) |
|  | *tet*(O)-R | TCCCACTGTTCCATATCGTCA |  |  |
| *tet*(S) | *tet*(S)-F | CATAGACAAGCCGTTGACC | 667 | In this study |
|  | *tet*(S)-R | ATGTTTTTGGAACGCCAGAG |  |  |
| *mepR* | *mepR*-F | catctaacgaaatggtggtgc | 475 | In this study |
|  | *mepR*-R | gactgatttctaccagtcacac |  |  |
| *mepA* | *mepA*-F | gtgtgactggtagaaatcagtc | 1481 | In this study |
|  | *mepA*-R | tactcagccagaagtggacg |  |  |
| *mepB* | *mepB*-F | CGTCCACTTCTGGCTGAGTA | 648 | In this study |
|  | *mepB*-R | AGTAGGACAAGAAACCGTTCA |  |  |
| *S3* | *S3*-F | ACGGTAAAGAAGAAGCTAAAG | 810 | (Bai et al., 2019) |
|  | *S3*-R | GCAGATTCGATTTGACGAGAT |  |  |
| *S10* | *S10*-F | TTCAGAAGATTTCTCAGTGATTACG | 1920 | (Bai et al., 2019) |
|  | *S10*-R | CTCGAAAATAGTTGAACTGACTAAG |  |  |
| Primers used for *mepB* cloning | | | | |
| *mepB* | *mepB*-prom-F | CGGAATTCatgttcgtccacttctggct(*EcoRI*) | 650 | In this study |
|  | *mepB*-prom-R | GCTCTAGAaggacaagaaaccgttcatc(*XbaI*) |  |  |
| Primers used for qRT-PCR | | | | |
| *gyrB* | *gyrB*-F | ACATTACAGCAGCGTATTAG | 111 | (Bai et al., 2019) |
|  | *gyrB*-R | CTCATAGTGATAGGAGTCTTCT |  |  |
| *mepA* | *mepA*-F | TTATGGAAACTTCGCGATTGC | 91 | (McAleese et al., 2005) |
|  | *mepA*-R | AACACCTTCACATAATCCCATGATAAT |  |  |
| *tet38* | *tet38*-F | TGACAGGTGTGGCTATTGGT | 112 | (Chen and Hooper, 2018) |
|  | *tet38*-R | TTGCCTGGGAAATTTAATGC |  |  |
| *tet*(K) | *tet*(K)-F | ACTGATTATGGTGGTTGTAG | 162 | In this study |
|  | *tet*(K)-R | ATAGGAAGTATAAGTAGGTAAGAC |  |  |
| *tet*(L) | *tet*(L)-F | AACCCAATTACCGACCCGAA | 150 | In this study |
|  | *tet*(L)-R | ACCTGCGAGTACAAACTGGG |  |  |

**Table S2 Efflux pump phenotype test for tetracyclines-resistant *Staphylococcus aureus* isolates**

| Isolates | MIC (μg/mL) | | fold changes | Efflux pump phenotype^a^ |  | MIC (μg/mL) | | fold changes | Efflux pump phenotype |  | MIC (μg/mL) | | fold  changes | Efflux pump phenotype |
| --- | --- | --- | --- | --- | --- | --- | --- | --- | --- | --- | --- | --- | --- | --- |
|  | OMC | OMC+CCCP (0.4 μg/mL) |  |  |  | ERV | ERV+CCCP  (0.4 μg/mL) |  |  |  | TGC | TGC+CCCP  (0.4 μg/mL) |  |  |
| JP3349 | 4^R^ | 1 | 4 | + |  | 4^R^ | 1 | 4 | + |  | ≥8^R^ | 2 | ≥4 | + |
| JP3936 | 2^R^ | 0.5 | 4 | + |  | 0.12 | ND | ND | ND |  | 0.25 | ND | ND | - |
| JP4041 | 4^R^ | 1 | 4 | + |  | 0.5 | ND | ND | ND |  | 0.5 | ND | ND | - |
| JP4051 | 2^R^ | 2 | 1 | - |  | 0.12 | ND | ND | ND |  | 0.5 | ND | ND | - |
| JP4063 | 2^R^ | 2 | 1 | - |  | 0.12 | ND | ND | ND |  | 0.5 | ND | ND | - |
| JP4091 | 4^R^ | 4 | 1 | - |  | 0.5 | ND | ND | ND |  | 0.5 | ND | ND | - |
| JP4092 | 4^R^ | 2 | 2 | - |  | 0.12 | ND | ND | ND |  | 0.5 | ND | ND | - |
| JP4108 | 4^R^ | 2 | 2 | - |  | 0.5 | ND | ND | ND |  | 0.5 | ND | ND | - |
| JP4113 | 4^R^ | 2 | 2 | - |  | 0.25 | ND | ND | ND |  | 0.5 | ND | ND | - |
| JP4169 | 4^R^ | 1 | 4 | + |  | 0.12 | ND | ND | ND |  | 0.5 | ND | ND | - |
| JP4174 | 4^R^ | 0.5 | 8 | + |  | 0.5 | ND | ND | ND |  | 0.5 | ND | ND | - |
| JP4212 | 8^R^ | 4 | 2 | - |  | 0.12 | ND | ND | ND |  | 0.5 | ND | ND | - |
| JP4234 | 8^R^ | 2 | 4 | + |  | 0.5 | ND | ND | ND |  | 0.5 | ND | ND | - |
| JP4238 | 4^R^ | 4 | 1 | - |  | 0.5 | ND | ND | ND |  | 0.5 | ND | ND | - |
| JP4298 | 4^R^ | 2 | 2 | - |  | 0.5 | ND | ND | ND |  | 1^R^ | 0.5 | 2 | - |
| JP4341 | 4^R^ | 4 | 1 | - |  | 0.5 | ND | ND | ND |  | 0.5 | ND | ND | - |
| JP4344 | 8^R^ | 4 | 2 | - |  | 0.5 | ND | ND | ND |  | 1^R^ | 0.25 | 4 | + |
| JP4403 | 2^R^ | 0.25 | 8 | + |  | 0.12 | ND | ND | ND |  | 0.5 | ND | ND | - |
| JP4474 | 4^R^ | 2 | 2 | - |  | 0.5 | ND | ND | ND |  | 0.5 | ND | ND | - |
| JP4499 | 4^R^ | 2 | 2 | - |  | 0.5 | ND | ND | ND |  | 0.5 | ND | ND | - |
| JP4612 | 4^R^ | 1 | 4 | + |  | 2^R^ | 0.5 | 4 | + |  | 2^R^ | 0.25 | 8 | + |
| JP4808 | 8^R^ | 4 | 2 | - |  | 0.5 | ND | ND | ND |  | 0.5 | ND | ND | - |
| JP4865 | 4^R^ | 4 | 1 | - |  | 0.5 | ND | ND | ND |  | 0.5 | ND | ND | - |
| JP4868 | 4^R^ | 2 | 2 | - |  | 0.5 | ND | ND | ND |  | 0.5 | ND | ND | - |
| JP4887 | 4^R^ | 2 | 2 | - |  | 0.5 | ND | ND | ND |  | 0.5 | ND | ND | - |
| JP4996 | 4^R^ | 2 | 2 | - |  | 0.5 | ND | ND | ND |  | 1^R^ | 0.5 | 2 | - |
| JP5013 | 2^R^ | 2 | 1 | - |  | 0.12 | ND | ND | ND |  | 0.25 | ND | ND | - |
| JP5054 | 4^R^ | 2 | 2 | - |  | 0.5 | ND | ND | ND |  | 0.5 | ND | ND | - |
| JP5068 | 4^R^ | 4 | 1 | - |  | 0.5 | ND | ND | ND |  | 0.5 | ND | ND | - |
| JP5113 | 8^R^ | 1 | 8 | + |  | 1^R^ | 0.125 | 8 | + |  | 1^R^ | 0.12 | 8 | + |
| JP5222 | 4^R^ | 4 | 1 | - |  | 0.5 | ND | ND | ND |  | 0.5 | ND | ND | - |
| JP5236 | 4^R^ | 4 | 1 | - |  | 0.5 | ND | ND | ND |  | 1^R^ | 0.5 | 2 | - |
| JP5265 | 4^R^ | 2 | 2 | - |  | 0.12 | ND | ND | ND |  | 0.5 | ND | ND | - |
| JP5482 | 4^R^ | 1 | 4 | + |  | 0.12 | ND | ND | ND |  | 1^R^ | 1 | 1 | - |
| JP5501 | 8^R^ | 4 | 2 | - |  | 0.5 | ND | ND | ND |  | 1^R^ | 0.5 | 2 | - |
| JP5631 | 4^R^ | 2 | 2 | - |  | 0.5 | ND | ND | ND |  | 0.5 | ND | ND | - |
| JP5732 | 8^R^ | 4 | 2 | - |  | 0.5 | ND | ND | ND |  | 0.5 | ND | ND | - |
| JP5743 | 4^R^ | 4 | 1 | - |  | 0.5 | ND | ND | ND |  | 0.5 | ND | ND | - |
| JP5872 | 4^R^ | 2 | 2 | - |  | 0.5 | ND | ND | ND |  | 0.5 | ND | ND | - |
| JP5965 | 4^R^ | 2 | 2 | - |  | 0.5 | ND | ND | ND |  | 0.5 | ND | ND | - |
| JP6053 | 2^R^ | 1 | 1 | - |  | 0.12 | ND | ND | ND |  | 0.5 | ND | ND | - |

MIC, minimum inhibitory concentration; ERV, eravacycline; OMC, omadacycline; TGC, tigecycline; CCCP, carbonyl cyanide m-chlorophenylhydrazone; Superscript “R” indicates resistance; “+” indicates the strains with positive efflux pump phenotype; “-” indicates the strains with negative efflux pump phenotype; ND means not detection; ^a^ Compared with ERV, OMC or TGC alone, the MIC value of ERV, OMC or TGC decreased 4-fold or more was confirmed to have an inhibitory effect when ERV, OMC or TGC were combined with 0.4 μg/mL CCCP.

**Table S3 Fold changes of genes expression in *Staphylococcus aureus* exposed to 1/2 MIC concentrations tetracyclines**

| Isolates | treated with 1/2 MIC tetracyclines | Fold changes of genes expression (mean *±* SD) | | | |
| --- | --- | --- | --- | --- | --- |
|  |  | *mepA* | *tet38* | *tet*(K) | *tet*(L) |
| JP3349 | eravacycline | 0.35 ± 0.02 | **2.94 ± 0.36** | **39.23 ± 3.07** | 0.60 ± 0.13 |
| JP4612 | eravacycline | **18.27 ± 0.29** | **3.90 ± 0.31** | **3.32 ± 0.24** | 0.76 ± 0.18 |
| JP5113 | eravacycline | 1.45 ± 0.29 | 1.16 ± 0.08 | **18.7 ± 1.64** | 0.16 ± 0.04 |
| JP3349 | omadacycline | 0.51 ± 0.01 | **2.11 ± 0.06** | **24.67 ± 0.81** | **2.51 ± 0.17** |
| JP3936 | omadacycline | 0.36 ± 0.02 | 0.39 ± 0.28 | 1.00 ± 0.02 | **3.33 ± 0.18** |
| JP4041 | omadacycline | **3.62 ± 0.38** | **1.80 ± 0.23** | **4.40 ± 0.19** | **1.92 ± 0.16** |
| JP4169 | omadacycline | **2.80 ± 0.12** | **2.39 ± 0.18** | **5.87 ± 0.16** | 1.13 ± 0.23 |
| JP4174 | omadacycline | 0.90 ± 0.02 | 1.26 ± 0.12 | **2.15 ± 0.05** | 1.26 ± 0.41 |
| JP4344 | omadacycline | 1.07 ± 0.05 | 0.99 ± 0.17 | **1.83 ± 0.17** | **4.59 ± 0.64** |
| JP4403 | omadacycline | 0.08 ± 0.01 | 0.30 ± 0.01 | 0.24 ± 0.00 | 0.46 ± 0.15 |
| JP4612 | omadacycline | **3.09 ± 0.13** | **5.27 ± 0.54** | **8.09 ± 0.13** | 0.58 ± 0.23 |
| JP5113 | omadacycline | **2.55 ± 0.09** | **2.63 ± 0.09** | **48.64 ± 3.56** | 1.58 ± 0.64 |
| JP5482 | omadacycline | 0.74 ± 0.22 | 0.93 ± 0.06 | 0.00 ± 0.00 | **3.23 ± 0.20** |
| JP3349 | tigecycline | **2.90 ±0.56** | **1.53 ±0.16** | **16.02 ± 1.80** | 0.57 ± 0.02 |
| JP4344 | tigecycline | 0.73 ± 0.00 | 0.07 ± 0.02 | **1.71 ± 0.06** | **6.48 ± 0.99** |
| JP4612 | tigecycline | **8.40 ± 0.94** | 1.55 ± 0.20 | **4.70 ± 0.04** | **5.17 ± 1.26** |
| JP5113 | tigecycline | **12.66 ± 1.28** | **7.22 ± 0.26** | **222.51 ± 6.06** | **4.27 ± 0.20** |

MIC, minimum inhibitory concentration; Bold indicates that the Fold changes of genes expression are statistically significant after exposed to 1/2 MIC concentrations tetracyclines compared with 0 MIC.

## Supplementary Figures


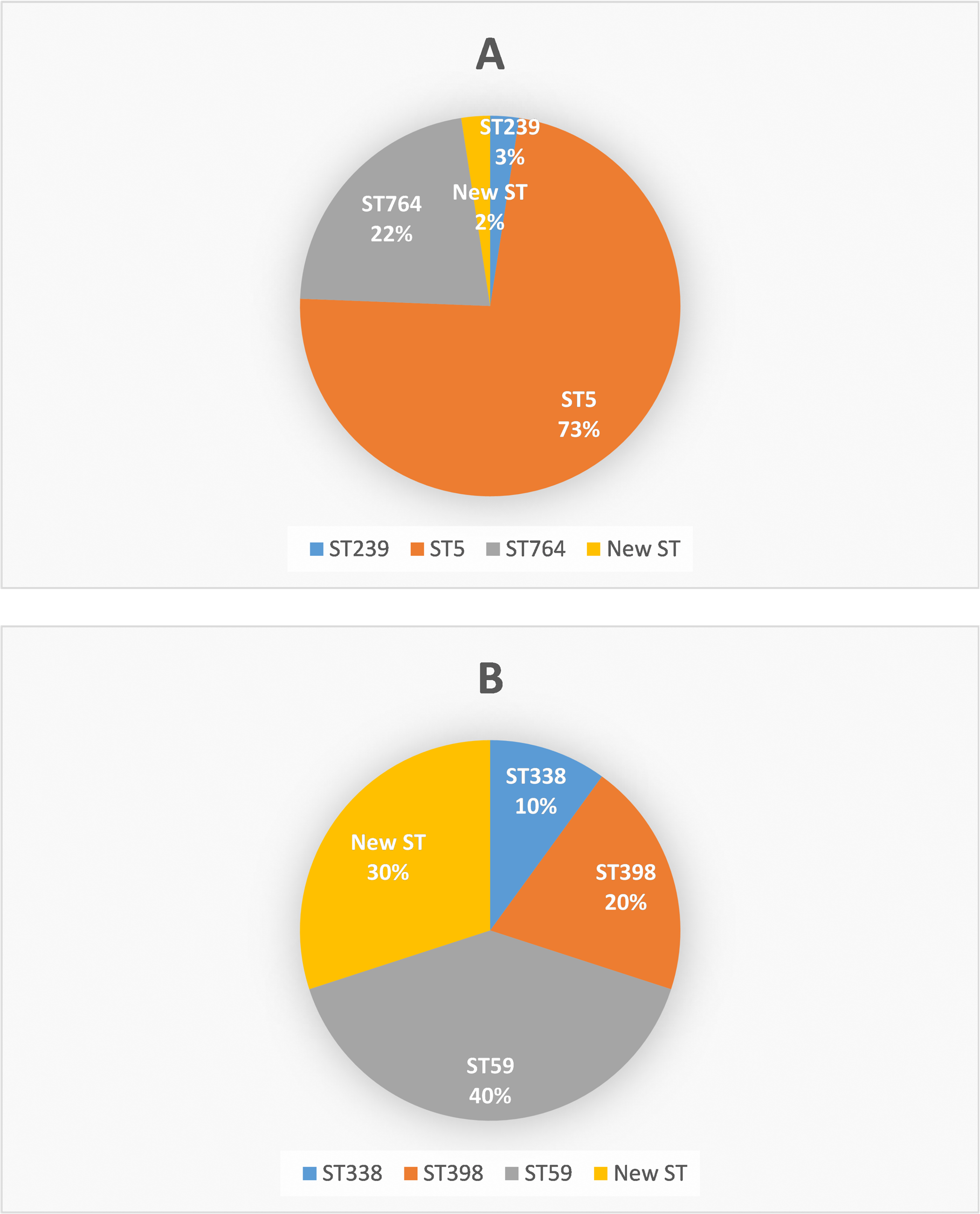


**Figure S1 ST distrubution of forty-one tetracyclines-resistant (A) and ten tetracyclines-susceptible (B) *Staphylococcus aureus* isolates.**

Bai, B., Lin, Z., Pu, Z., Xu, G., Zhang, F., Chen, Z., et al. (2019). In vitro Activity and Heteroresistance of Omadacycline Against Clinical Staphylococcus aureus Isolates From China Reveal the Impact of Omadacycline Susceptibility by Branched-Chain Amino Acid Transport System II Carrier Protein, Na/Pi Cotransporter Family Protein, and Fibronectin-Binding Protein. *Front Microbiol* 10**,** 2546. doi: 10.3389/fmicb.2019.02546.

Chen, C., and Hooper, D.C. (2018). Effect of Staphylococcus aureus Tet38 native efflux pump on in vivo response to tetracycline in a murine subcutaneous abscess model. *J Antimicrob Chemother* 73(3)**,** 720-723. doi: 10.1093/jac/dkx432.

McAleese, F., Petersen, P., Ruzin, A., Dunman, P.M., Murphy, E., Projan, S.J., et al. (2005). A novel MATE family efflux pump contributes to the reduced susceptibility of laboratory-derived Staphylococcus aureus mutants to tigecycline. *Antimicrob Agents Chemother* 49(5)**,** 1865-1871. doi: 10.1128/AAC.49.5.1865-1871.2005.

**References**
